# 6-Shogaol Abrogates Parkinson’s Disease in Rotenone-Induced Rodents: Based on In Silico Study and Inhibiting TNF-α/NF-κB/IL-1β/MAO-B

**DOI:** 10.3390/ph17101348

**Published:** 2024-10-09

**Authors:** Misbahuddin Rafeeq, Fahad A. Al-Abbasi, Muhammad Afzal, Ehssan Moglad, Salwa D. Al-Qahtani, Sami I. Alzrea, Naif A. R. Almalki, Faisal Imam, Nadeem Sayyed, Imran Kazmi

**Affiliations:** 1 Department of Pharmacology Faculty of Medicine, Rabigh King Abdulaziz University, Jeddah 21589, Saudi Arabia; 2 Department of Biochemistry, Faculty of Sciences, King Abdulaziz University, Jeddah 21589, Saudi Arabia; 3 Department of Pharmaceutical Sciences, Pharmacy Program, Batterjee Medical College, Jeddah 21442, Saudi Arabia; mohmmad.afzal@bmc.edu.sa; 4 Department of Pharmaceutics, College of Pharmacy, Prince Sattam bin Abdulaziz University, Alkharj 11942, Saudi Arabia; e.moglad@psau.edu.sa; 5 Department of Medical Laboratory Sciences, College of Applied Medical Sciences, Majmaah University, Al Majmaah 11952, Saudi Arabia; 6 Department of Pharmacology, College of Pharmacy, Jouf University, Aljouf, Sakaka 72341, Saudi Arabia; samisz@ju.edu.sa; 7 Experimental Biochemistry Unit, King Fahd Medical Research Center, King Abdulaziz University, Jeddah 21589, Saudi Arabia; 8 Department of Pharmacology and Toxicology, College of Pharmacy, King Saud University, Riyadh 11451, Saudi Arabia; fimam@ksu.edu.sa; 9 School of Pharmacy, Glocal University, Saharanpur 247121, India

**Keywords:** 6-shogaol, neuroinflammation, neuroprotective, rotenone, Parkinson’s disease

## Abstract

**Background/Objectives:** 6-Shogaol is a comparatively innovative anti-Parkinson’s remedy with antioxidant and anti-inflammatory characteristics. This investigation intended to determine the role of 6-shogaol in the Parkinson’s disease (PD) paradigm in rotenone-induced rats. **Methods:** Thirty male Wistar rats (10–12 weeks old; 180 ± 20 g) were divided into five groups. Animals with rotenone-induced experimental PD were subsequently treated with 6-shogaol-10 at 20 mg/kg for 28 days. After the experimental duration, behavioural investigations were performed, i.e., open field test, forced swim test, rotarod test, and catalepsy test. Biochemical assessments like AChE, GSH, CAT, SOD, MDA, nitrite, ceruloplasmin, proinflammatory markers such as IL-1β, NF-κB, TNF-α, and catecholamines markers (DA, GABA, and MAO-B) were determined. The docking procedure was conducted using the AutoDock Vina docking protocol. Furthermore, histopathology was performed. **Results:** Rotenone significantly increased the level of MAO-B, oxidative, nitrative, and pro-inflammatory markers. However, there was a decline in ceruloplasmin, dopamine, and endogenous antioxidants. Treatment with 6-shogaol (10 and 20 mg/kg) considerably sustained the elevation of oxidative stress and inflammatory indicators and decreased AChE activity and dopamine levels. In the histology of the brain, 6-shogaol improved the neuronal structure and reduced the degeneration of neurons. Based on the binding energy values, compound 6-shogaol demonstrates a favourable binding affinity to AChE, MAO-B, DA, and GABA with respective binding energies of −8.214, −8.133, −7.396 and −6.189 kcal/mol. **Conclusions:** In this study, 6-shogaol exhibited neuroprotective properties against PD, which could be employed as a prospective medication for PD.

## 1. Introduction

The neurodegenerative disorder Parkinson’s disease (PD) is identified by the destruction of the dopaminergic neurons in the area of the brain, specifically the nigrostriatal region. It is related to various clinical afflictions, including tremors, bradykinesia, tight muscles, poor posture and stability, loss of spontaneous activities, changes in tongue and writing, and loss of automatic movements [1]. In this disorder, dopamine (DA) in the striatum is downregulated [2], and acetylcholine (Ach) and DA levels are imbalanced due to neurodegeneration of the dopaminergic system [3,4,5]. There is no cure for PD, which is affected by the drop of DA neurotransmitters in the substantia nigra pars compacta (SNpc) [6]. The DA neurons may play an imperative role in various brain functions including behavioral and voluntary behavior [7]. To explain PD, there are several components: gross manifestation, neural death, cellular pathology, molecular mechanisms that lead to progressive degeneration, and genetic and environmental dysregulation of proteins that lead to aggregated α-synuclein inclusions within cells [8]. Several paradigms have been employed to investigate PD in rodents. Among these, the rotenone model suggests that common mechanisms link genetic and sporadic forms of PD [8] and serve as a conventional mitochondrial complex I inhibitor of the mitochondrial respiratory chain. In experimental animals, rotenone induces symptoms of PD similar to those seen in humans when exposed to this neurotoxin [7]. Interestingly, rotenone replicates many symptoms of human idiopathic Parkinson’s in rodents, comprising abnormal body functioning and pathological brain alterations such as nigrostriatal degeneration and the inclusion of synuclein. After rotenone exposure, rodents can show progressive nigral degeneration and behavioral deficits [7,8,9]. Rotenone acts via induction of oxidative stress and subsequent loss of dopaminergic neurons in rats [6]. Rotenone exposure also resulted in a significant upregulation of pro-inflammatory cytokines, including interleukin-1β (IL-1β) and tumor necrosis factor-α (TNF-α). Concomitantly, rotenone decreased the enzymatic activities of superoxide dismutase (SOD) and catalase and reduced glutathione levels (GSH). The combined effect of increased reactive oxygen species (ROS) production and decreased antioxidant capacity led to oxidative stress, a key mediator of rotenone-induced cellular damage [6,9,10]. Rotenone has been used as a model for PD in recent years to examine the mechanism of cell death and possible therapeutic involvement [10,11]. The available drugs for the treatment of PD have various side effects (nausea, depression, hallucinations, convulsions, anxiety, peripheral vasospasm) [12]. DA replacement therapy is currently used for treating PD, but it can cause long-term complications, including dyskinesia. Neurodegenerative disorders like Parkinson’s can be treated with herbal or plant remedies that are effective and safer than drugs [13]. Hence, herbal medicines are considered complementary remedies as they carry attentive curative actions [13]. Rhizomes of *Zingiber officinale* L., usually known as ginger, play an essential role in culinary preparation and medical procedures [14]. A main reactive component in dried ginger, 6-shogaol has conventionally been used for treating an extensive range of ailments. In addition to its pungent flavor, 6-gingerol, which is found in fresh ginger before dehydration, is also reported to be responsible for its pungent flavor [15]. 6-Shogaol, an active composite of ginger, has revealed the traumatic brain injury-induced anxiety/depression and anti-inflammatory mechanism in rodents [16]. It exhibits antioxidant, anti-inflammatory, anticancer, and antiemetic properties. Additionally, it offers potential health benefits, including cardiovascular and neuroprotective effects [15]. Previous studies demonstrated that 6-shogaol exerts a potent inhibitory effect on cell migration and proliferation. 6-shogaol inhibits the nuclear factor kappa B (NF-kB) p65 pathway by preventing IkB-α from being phosphorylated and degraded, which in turn prevents pro-inflammatory indices with JNK regulation. 6-shogaol can also inhibit the production of pro-inflammatory cytokines such as interleukin-1β and tumor necrosis factor-α. Furthermore, it has been shown to reduce inflammation and oxidative stress in in vitro and in vivo studies. 6-Shogaol acts as a neuroprotective, anti-inflammatory, antioxidant, anticancer, gastroprotective, and analgesic agent in different animal models [15,16]. Additionally, Studies demonstrate that 6-shogaol possesses the ability to mitigate β-amyloid-induced neuroinflammation and cognitive deficits by suppressing the activation of glial cells in murine models [16]. It shields dopaminergic neurons in PD models via anti-neuroinflammation [17]. This research aims to determine whether 6-shogaol prevents the development of PD in rodents induced by rotenone in Wistar rats based on the behavioral paradigms (open field test, forced swim test, rotarod test, and catalepsy test), biochemical parameters (Oxidative stress markers, pro-inflammatory cytokines, neurotransmitter levels, and AChE), histopathological examination and molecular docking. Molecular docking was employed with the aim of evaluating the binding affinities of the selected compounds to the target proteins. 

## 2. Results

### 2.1. Effect of 6-Shogaol on Behavioral Paradigms

#### 2.1.1. Effect of 6-Shogaol on Open Field Test

**Figure 1:**
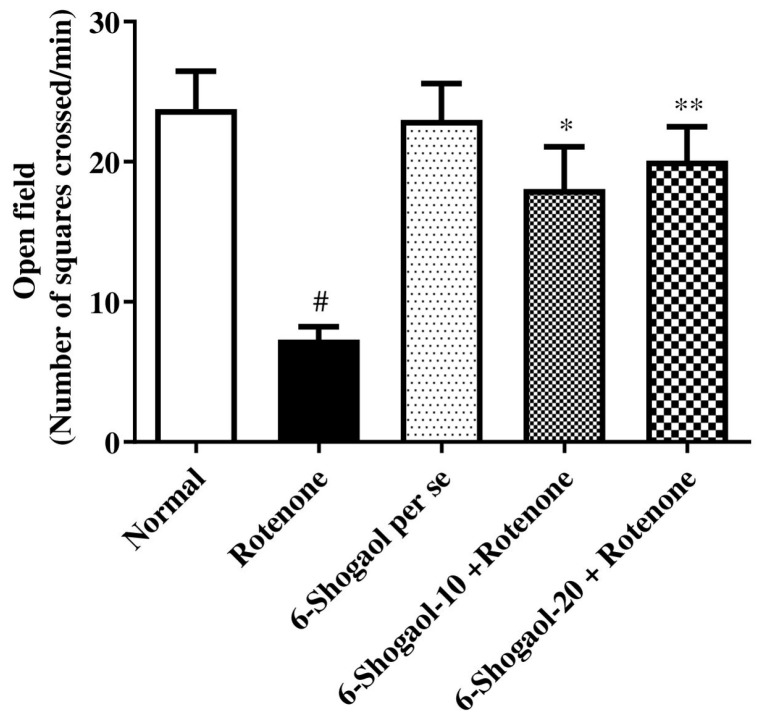
Effect of 6-shogaol on open field test in rotenone-treated rats. Values are expressed as mean ± SEM. A comparison amongst the groups was done using Tukey’s post hoc test using one-way ANOVA. *p* < 0.01, 0.001 were expressed as *, **, respectively. # Significant as correlated to the control group (*p* < 0.0001).

The locomotor activity, including rearing, grooming, and square crossings, was considerably reduced after rotenone treatment compared to normal control (*p* < 0.0001). In comparison with rotenone control, 6-shogaol at doses 10 and 20 mg/kg exhibits considerable [F (4, 25) = 7.337, (*p* = 0.0005)] and improved motor function as reflected by the number of squares crossed. The 6-shogaol per se group showed no substantial changes (Figure 1). 

#### 2.1.2. Effect of 6-Shogaol on Forced Swim Test

**Figure 2:**
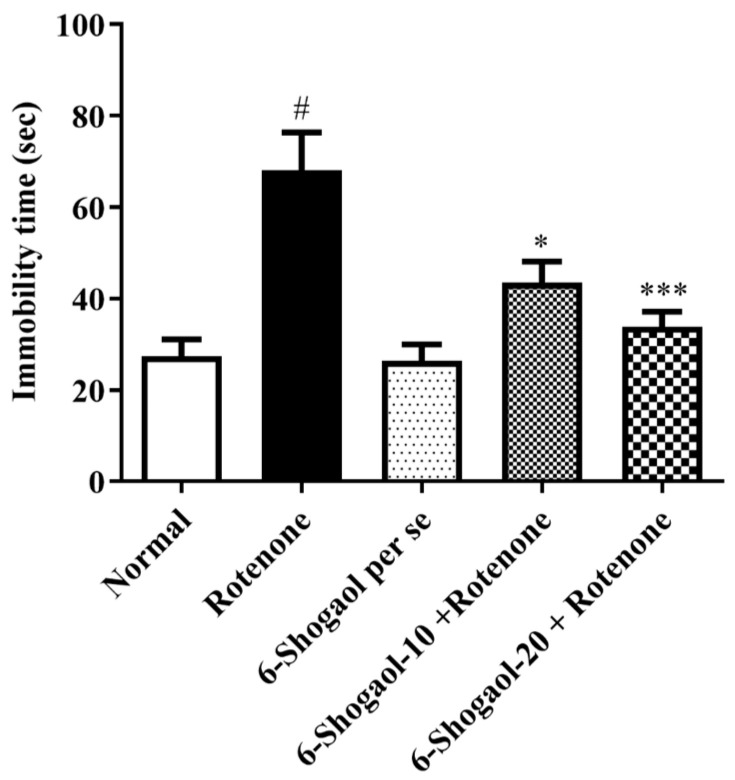
Effect of 6-shogaol on forced swim test in rotenone-treated rats. Values are expressed as mean ± SEM. A comparison amongst the groups was done using Tukey’s post hoc test using one-way ANOVA. *p* < 0.01, 0.0001 were expressed as *, ***, respectively. # Significant as correlated to the control group (*p* < 0.0001).

Group I expended shorter periods in an immovable state than group II (*p* < 0.0001). Rotenone administration caused despair and spent considerable time in an immovable state in the forced swim test. Rats given both doses (10 and 20 mg/kg) of 6-shogaol spent less time in the immovable state [F (4, 25) = 11.66, (*p* < 0.0001)] correspondingly, associated to rats treated with rotenone. Per se, the 6-shogaol group did not reveal any notable changes. The result of the forced swim test is displayed in Figure 2.

#### 2.1.3. Effect of 6-Shogaol on Rotarod Test

**Figure 3:**
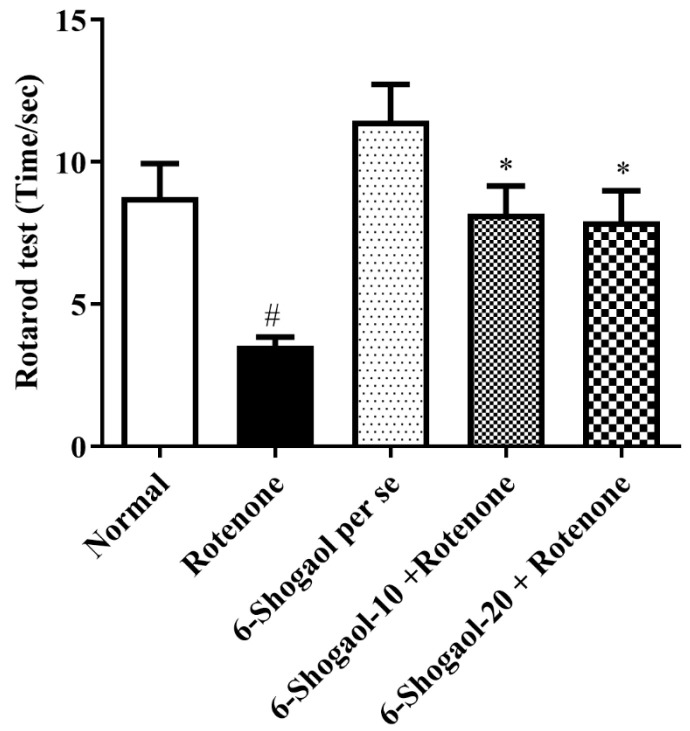
Effect of 6-shogaol on rotarod test in rotenone-treated rats. Values are expressed as mean ± SEM. A comparison amongst the groups was done using Tukey’s post hoc test using one-way ANOVA. *p* < 0.01 were expressed as *, respectively. # Significant as correlated to the control group (*p* < 0.0001).

Figure 3 represents the consequences of 6-shogaol on rotenone convinced changes in the falling time of animals. Falling time was significantly decreased in the rotenone control group compared to the normal group (*p* < 0.0001). Rats treated with 6-shogaol-10 and 20 mg/kg showed substantial [F (4, 25) = 7.841, (*p* = 0.0003)] improvement in falling time as associated with the rotenone-treated group. 6-Shogaol per se group did not exhibit any notable alterations.

#### 2.1.4. Effect of 6-Shogaol on Catalepsy

**Figure 4:**
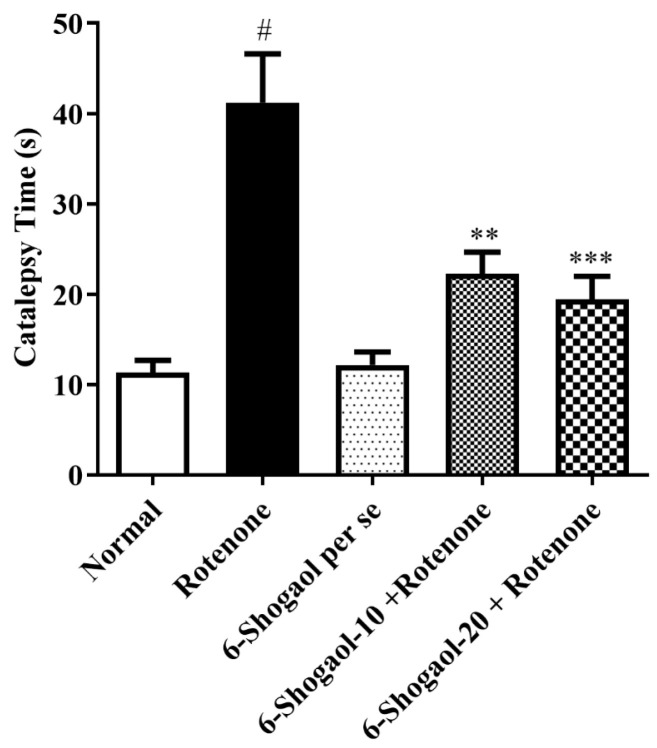
Effect of 6-shogaol on catalepsy test in rotenone-treated rats. Values are expressed as mean ± SEM. A comparison amongst the groups was done using Tukey’s post hoc test using one-way ANOVA. *p* < 0.001, 0.0001 were expressed as **, *** respectively. # Significant as correlated to the control group (*p* < 0.0001).

An analysis of post-hoc data indicates that rats treated with rotenone enhanced cataleptic behavior compared to normal control rats (*p* < 0.0001). A significant improvement in latency was detected for animals treated with rotenone to remove their paw from the higher surface associated with normal control animals. Animals treated with 6-shogaol (10 and 20 mg/kg) significantly enhanced muscular inflexibility and significantly decreased [F (4, 25) = 16.23, (*p* < 0.0001)] correspondingly, time to take out a paw from the higher surface compared to rotenone group (Figure 4). No significant alterations were found in the 6-shogaol per se group.

### 2.2. Biochemical Estimations

#### Effect of 6-Shogaol on AChE Activity

**Figure 5:**
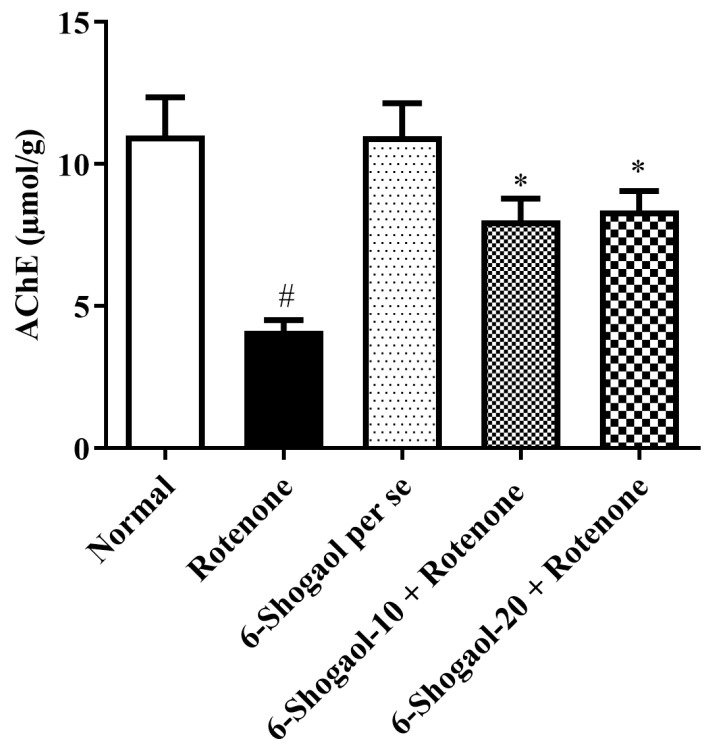
Effect of 6-shogaol on AChE activity in rotenone-treated rats. Values are expressed as mean ± SEM. A comparison amongst the groups was done using Tukey’s post hoc test using one-way ANOVA. *p* < 0.01 were expressed as * respectively. # Significant as correlated to a control group (*p* < 0.0001).

When associated with the normal group, the rotenone group exhibits a reduction in AChE (*p* < 0.0001). In contrast to the rotenone group, treatment with 6-shogaol-10 and 20 mg/kg produced significant [F (4, 25) = 9.117, (*p* = 0.0001)] increased the AChE level (Figure 5). No significant changes were observed in the 6-shogaol per se group. 

### 2.3. Antioxidant Levels

#### 2.3.1. Effect of 6-Shogaol on GSH Level

**Figure 6:**
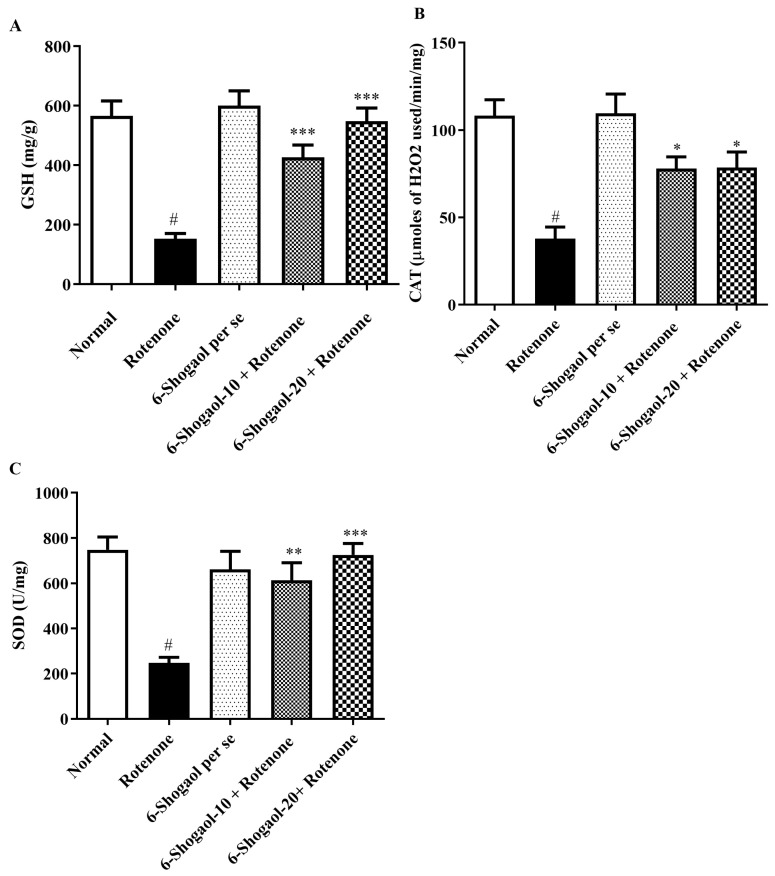
(**A**–**C**) Effect of 6-Shogaol on endogenous antioxidant levels in rotenone-treated rats. (**A**) GSH, (**B**) CAT, (**C**) SOD. Values are expressed as mean ± SEM. Comparison amongst the groups was done using Tukey’s post hoc test by one-way ANOVA. *p* < 0.01, 0.001, 0.0001 were expressed as *, **, *** respectively. # Significant as correlated to the control group (*p* < 0.0001).

The level of GSH declined in the rotenone group as associated with the normal group (*p* < 0.0001). A significant [F (4, 25) = 18.90, (*p* < 0.0001)] elevation in GSH level was observed in the 6-shogaol-treated groups (10 and 20 mg/kg) when associated with the rotenone group (Figure 6A). No appreciable changes were seen in the 6-shogaol per se group.

#### 2.3.2. Effect of 6-Shogaol on CAT Level

The CAT level decreased in the rotenone group as associated with the normal group (*p* < 0.0001). Significant [F (4, 25) = 11.40, (*p* < 0.0001)] increased CAT levels were detected in the brain homogenates of 6-shogaol at doses of 10 and 20 mg/kg in contrast to the rotenone group. 6-Shogaol per se did not exhibit any notable alterations (Figure 6B).

#### 2.3.3. Effect of 6-Shogaol on SOD Level

Group I showed elevation in the SOD level as associated with the rotenone group (*p* < 0.0001). 6-Shogaol-treated groups with lower (10 mg/kg) and higher doses (20 mg/kg) significantly restored [F (4, 25) = 10.97, (*p* < 0.0001)] the levels of SOD towards normal when associated with the rotenone group. 6-Shogaol per se 20 mg/kg showed no notable modifications (Figure 6C).

### 2.4. MDA and Nitrite Oxide Level

#### 2.4.1. Effect of 6-Shogaol on MDA Level

**Figure 7:**
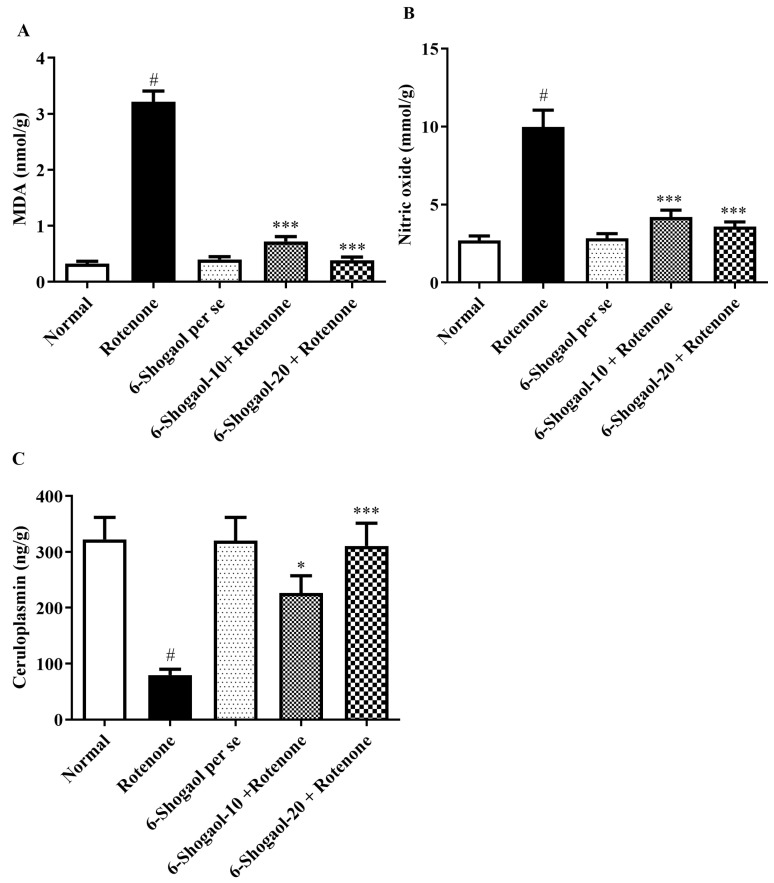
(**A**–**C**) Effect of 6-shogaol on oxidative and nitrative stress markers in rotenone-treated rats. (**A**) MDA, (**B**) Nitric oxide, (**C**) Ceruloplasmin. Values are expressed as mean ± SEM. Comparison amongst the groups was done using Tukey’s post hoc test by one-way ANOVA. *p* < 0.01, 0.0001 were expressed as *, *** respectively. # Significant as correlated to the control group (*p* < 0.0001).

The rotenone group revealed an elevation in brain MDA concentrations as compared with group I (*p* < 0.0001). Treatment with a 6-shogaol-treated group (10 and 20 mg/kg) significantly attenuated the increased amounts of MDA [F (4, 25) = 153.1, (*p* < 0.0001)] when associated with the rotenone group. No remarkable alterations were observed in the 6-shogaol per se group (Figure 7A).

#### 2.4.2. Effect of 6-Shogaol on Nitrite Oxide Level

Rats treated with rotenone displayed an increase in brain nitrite concentrations compared with normal control animals (*p* < 0.0001). 6-Shogaol treated group (10 and 20 mg/kg) significantly attenuated the increased amounts of nitrite [F (4, 25) = 28.36, (*p* < 0.0001)] when associated with the rotenone group. 6-Shogaol (per se 20 mg/kg) did not show any considerable changes (Figure 7B).

#### 2.4.3. Effect of 6-Shogaol on CP Level

Rotenone administration caused a substantial decline in CP level as associated with the control group (*p* < 0.0001). Conversely, 6-Shogaol-10 and 20 mg/kg produced a significant [F (4, 25) = 8.992, (*p* = 0.0001)] increase in CP level as associated with the rotenone group. No changes were noticed in the per se group (Figure 7C).

### 2.5. Effect of 6-Shogaol on Proinflammatory Levels

**Figure 8:**
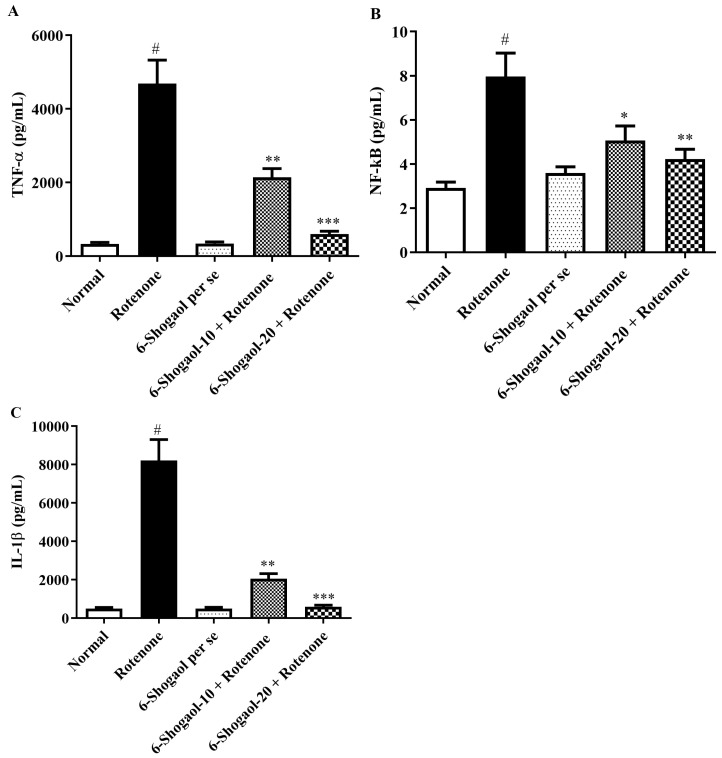
(**A**–**C**) Effect of 6-shogaol on proinflammatory markers in rotenone-treated rats. (**A**) TNF-α, (**B**) NF-κB, (**C**) IL-1β. Values are expressed as mean ± SEM. Comparison amongst the groups was done using Tukey’s post hoc test by one-way ANOVA. *p* < 0.01, 0.001, 0.0001 were expressed as *, **, *** respectively. # Significant as correlated to the control group (*p* < 0.0001).

Level of proinflammatory indicators significantly inhibited by 6-shogaol treatment. IL-1β, TNF-α, and NF-κB levels were considerably improved in rotenone-treated animals while associated with group I (*p* < 0.0001). Whereas 6-shogaol-10 and 20 mg/kg significantly lessened the levels of TNF-α [F (4, 25) = 37.71, (*p* < 0.0001)], NF-κB [F (4, 25) = 9.917, (*p* < 0.0001)] and IL-1β [F (4, 25) = 43.74, (*p* < 0.0001)] associated with the rotenone group (Figure 8A–C).

### 2.6. Effect of 6-Shogaol on Neurochemical Markers

**Figure 9:**
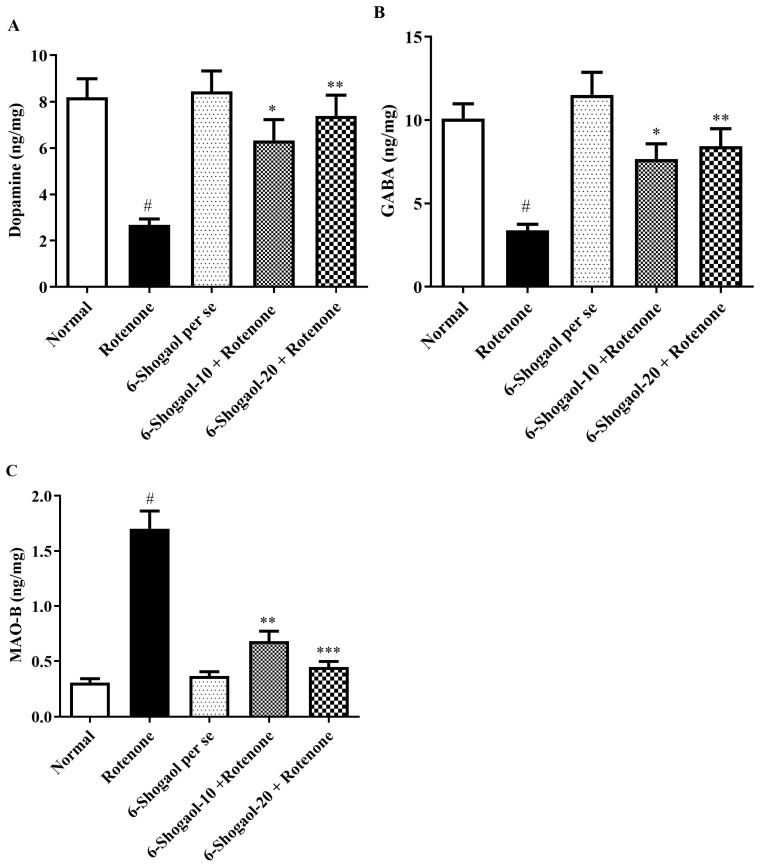
(**A**–**C**) Effect of 6-shogaol on neurochemical markers in rotenone-treated rats. (**A**) DA, (**B**) GABA, (**C**) MAO-B. Values are expressed as mean ± SEM. Comparison amongst the groups was done using Tukey’s post hoc test by one-way ANOVA. *p* < 0.01, 0.001, 0.0001 were expressed as *, **, *** respectively. # Significant as correlated to the control group (*p* < 0.0001).

The level of neurotransmitters markers (DA and GABA) in the rotenone-treated group decreased significantly as collated with the controls except for MAO-B level that shows elevation in the rotenone-treated animals (*p* < 0.0001). While the 6-shogaol treated group (10 and 20 mg/kg) exhibited a statistically significant rise in the DA [F (4, 25) = 8.953, (*p* = 0.0001)] and GABA [F (4, 25) = 10.27, (*p* < 0.0001)] levels when correlated to the rotenone group, whereas MAO-B level decreased [F (4, 25) = 42.12, (*p* < 0.0001)] as compared to rotenone group (Figure 9A–C). 

### 2.7. Effects of 6-Shogaol on Brain Tissue

**Figure 10:**
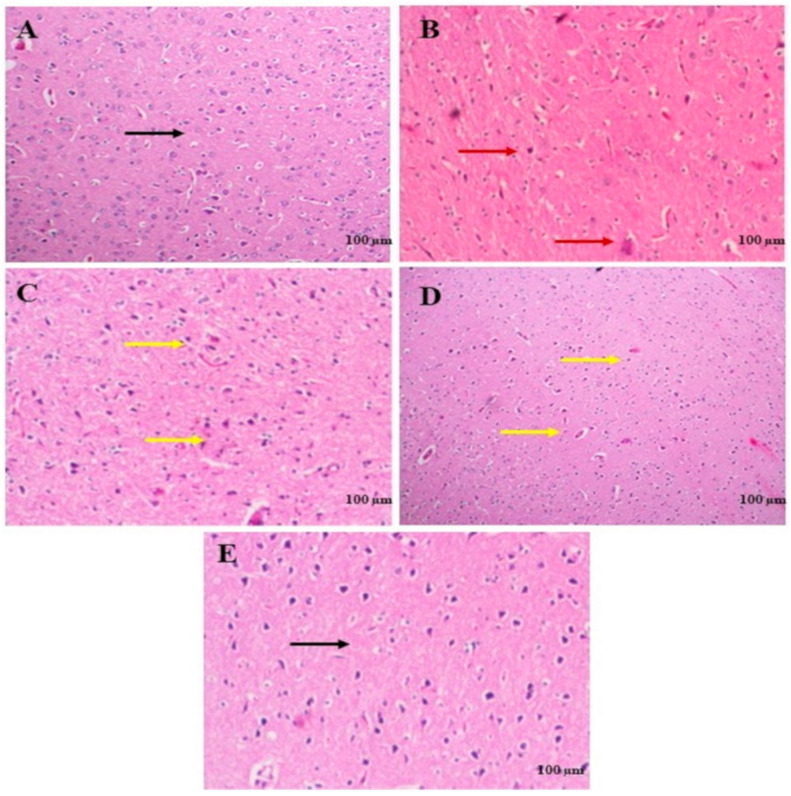
Effect of 6-shogaol on histopathological changes in rotenone-treated rats. (**A**) Normal group, (**B**) Rotenone induced group, (**C**) 6-shogaol Per se, (**D**) 6-shogaol (10 mg/kg), (**E**) 6-shogaol (20 mg/kg). The black arrows indicate normal neuronal cell; red arrows indicate neuronal damage; yellow arrows indicate-deeply-stained neuronal cells.

Figure 10 illustrates the neuroprotective effects of 6-shogaol on brain tissue, utilizing samples of brain tissue H and E staining. The detrimental effects of rotenone on brain tissue resulted in neuronal degeneration. Rats treated with 6-shogaol per se showed normal brain tissue. 6-shogaol-10 mg/kg ameliorated the rotenone effects as minor deeply stained neurons were observed, and more potent action occurred with the 6-shogaol-20 mg/kg in the brain histology.

### 2.8. Molecular Docking of the 6-Shogaol

**Figure 11:**
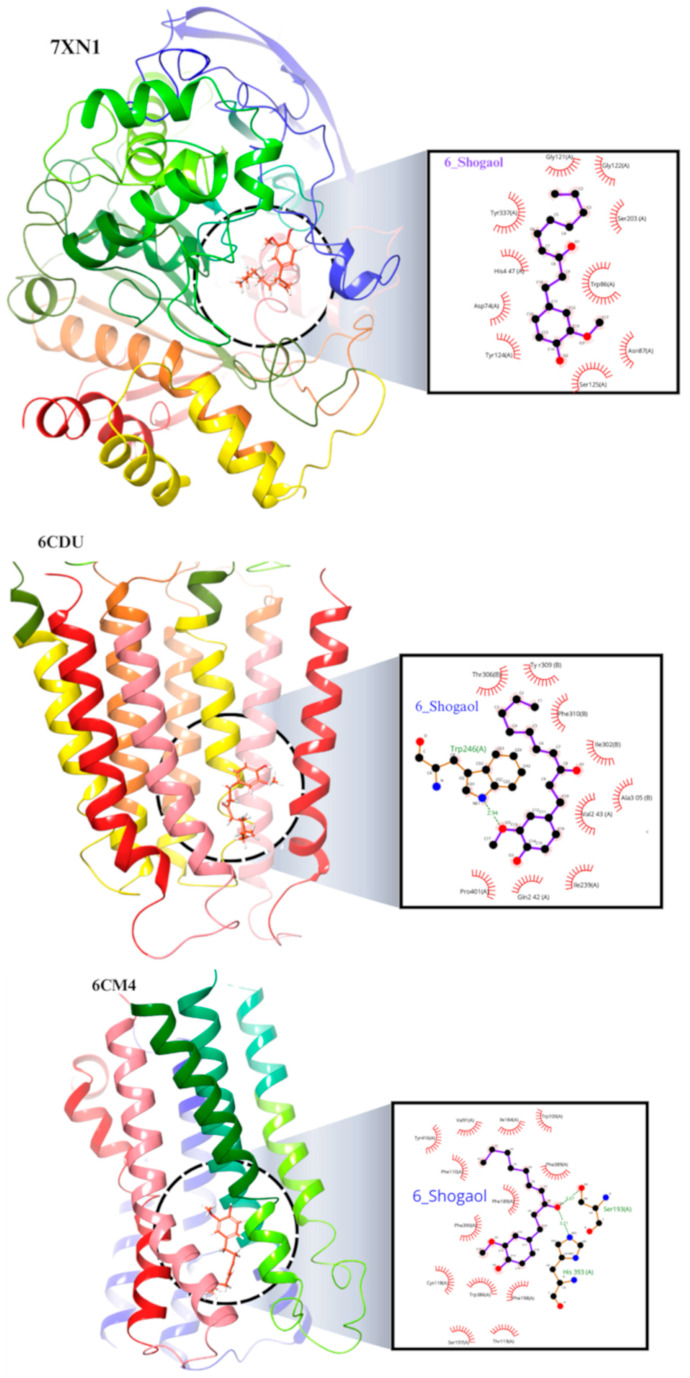

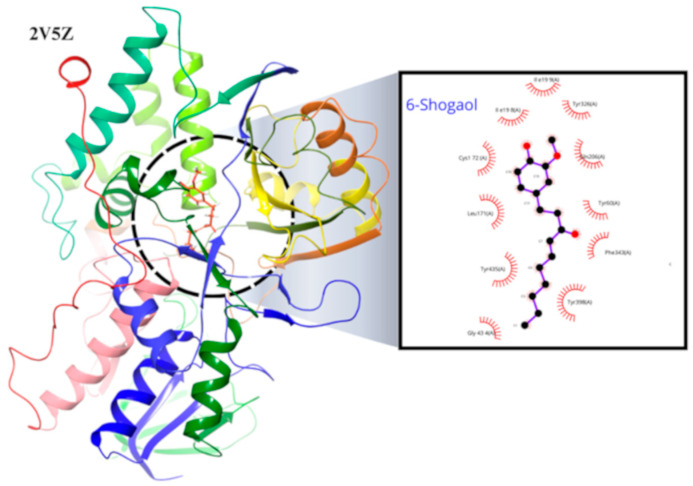
Molecular docking 2D and 3D images of the 6-shogaol with AChE (7XN1), Dopamine (6CM4), GABA (6CDU), and MAO-B (2V5Z). Proteins using LigPlot v1.4.5 Maestro V12.8 software.

Based on the binding energy values as represented in Table 1, compound 6-shogaol demonstrates a favorable binding affinity to AChE and MAO-B, with respective binding energies of −8.214 and −8.133 kcal/mol. This suggests that 6-shogaol may potentially act as an inhibitor of these enzymes, which are both responsible for breaking down Ach and monoamine neurotransmitters. In contrast, 6-shogaol exhibits a lower binding affinity to DA and GABA, with binding energies of −7.396 and −6.189 kcal/mol, respectively. This suggests that the compound may have a weaker interaction with these receptors, which modulate the activity of DA and GABA neurotransmitters, respectively (Figure 11).

## 3. Discussion

This research demonstrated that 6-shogaol was evaluated for anti-Parkinson’s effects in lessening neurodegeneration prompted in rats’ brains by systemic rotenone injection. One of the incurable disorders, PD is characterized by a deficit of neurotransmitter (DA) in SNpc. Secondly, drug-induced PD occurs when parkinsonism is caused by a known agent. This can lead to erroneous Parkinson’s diagnosis [10,18]. This study shows that 6-shogaol reduced the behavioral and biochemical aberrations caused by rotenone in rats.

In the evaluation of Parkinson’s treatment, rats’ paradigms have been developed, and they have proven to be quite successful. Rotenone is demonstrated to imitate the clinical, biochemical, and behavioral alterations associated with PD [8]. The current study showed a deterioration in behavioral patterns, antioxidant status, and neuroinflammatory markers in rotenone-induced PD for 28 days. Previously reported the mechanisms underlying dopaminergic damage brought on by oxidative stress, rotenone-induced PD is a good constructive paradigm [19]. Performance was measured in both the detriment of motor skill and motor coordination.

6-Shogaol showed improved performance on the rotarod and reduced immobility time and cataleptic symptoms, indicating that the compound helps preserve motor function and mitigates motor impairment, as demonstrated in Figure 1, Figure 2, Figure 3 and Figure 4. Compatible with reported similar results in the open-field test and lesser DA level in the brain. Moreover, rats injected with rotenone express poor movability, reduced falling time, loss of nigrostriatal dopaminergic neurons, and depletion in striatal DA [20]. However, our study showed that therapy with 6-shogaol creates a progressive improvement in this behavioral activity.

The etiology of PD is significantly influenced by the oxidative stress brought on by mitochondrial malfunction, particularly mitochondrial complex I impairment [21]. The evolution of ROS is the mitochondria, and when they are dysfunctional, the tissues suffer oxidative damage [22]. The current investigation revealed that the rotenone caused changes in all the biochemical parameters, including SOD, GSH, MDA, CAT, nitrite, CP, and AChE, which were all reversed by 6-shogaol at both doses.

Emerging evidence suggests a correlation between reduced AChE activity and the pathogenesis of various neurodegenerative disorders, including PD [23]. The neurotransmitter Ach is hydrolyzed by the cholinesterase family of enzymes; as an outcome, suppression of enzyme activity will increase the quantity of Ach in the brain. The loss of dopaminergic neurons in the substantia nigra and striatum has led to a disproportion in dopaminergic and cholinergic neurotransmission [24,25]. The drop-in AChE activity in the brains of rotenone-treated rats may represent the destruction of cholinergic neurons. In addition, the enzyme AChE is also sensitive to radical substance formation and inhibits its activity [26]. As demonstrated in Figure 5, the 6-shogaol treatment restored AChE activity and several of the deficiencies caused by rotenone in PD to almost the same level as normal controls. 6-Shogaol inhibits AChE activity, which could lead to increased levels of Ach, potentially improving cognitive function and motor control. Compared to previous studies conducted by Altharawi et al. and Fikry et al., our results are consistent with them [25,26].

In the present study, rotenone-induced rats substantially elevated MDA and nitrite, and reduced SOD, GSH, and CAT levels versus the normal group. The raised levels of MDA and nitrite have been detected in the PD [27]. Furthermore, nitric oxide has been revealed to suppress significant enzymes of energy metabolism that include the provoked formation of ROS, inducing even more harm to cellular mechanisms [28]. 6-Shogaol treatment reduces nitrite levels, indicating a decrease in nitric oxide production (Figure 7B). This reduction is likely due to its antioxidant and anti-inflammatory properties, which mitigate oxidative stress and downregulate the pathways involved in nitric oxide synthesis, thus protecting neurons from NO-mediated damage [15].

SOD level decreased in the rotenone-treated group, indicating the development of oxidative stress. Similar GSH depletion has been detected in the nigra of PD sufferers who have been determined with incidental Lewy bodies, which is now recognized as a preclinical indication of PD [13,29]. An enzyme-based antioxidant called CAT aids in reducing the toxicity of hydrogen peroxide. Oxidation stress causes the CAT level to decline. It has been noted that rotenone causes a decline in CAT levels, and this enzyme activation was thought to be a result of the brain’s built-in antioxidant resistance system [13]. The administration of 6-shogaol at both low and high doses significantly reduced MDA and nitrite levels, as evidenced by Figure 7A, B. Concurrently, there was a notable increase in the levels of GSH, CAT, and SOD, as illustrated in Figure 6C. These findings suggest that 6-shogaol treatment effectively enhanced the antioxidant capacity of the studied system. 6-Shogaol appears to decrease MDA level, a marker of lipid peroxidation, suggesting its ability to protect cells from oxidative damage. It may influence the activity of antioxidant enzymes like SOD and CAT, helping to neutralize harmful free radicals. The results of the current study are in accordance with a previous study by Shahid et al. and Fikry et al. [22,26].

PD is also characterized by iron accretion in the CNS and oxidative stress. CP controls cellular iron loading and export, preventing tissues from suffering oxidative damage [30]. Investigations on 6-shogaol’s antioxidant activity also focused on nonenzymatic antioxidants, or the second line of defense against oxidative stress, like ceruloplasmin, which scavenges residual free radicals that avoid being destroyed by antioxidant enzymes. Thus, CP appears necessary for regular iron transmission from cells to plasma. The in vitro discovery that CP performs as an enzyme (ferroxidase) that catalyzes the oxidation of ferrous iron served as the foundation for studies intended to describe the mechanism of CP action [31]. Our study showed that the rotenone group’s brain CP content dramatically decreased. The antioxidant impact of 6-shogaol at both doses was found to be a marked improvement in the activity of the CP marker, as depicted in Figure 7C. By increasing ceruloplasmin, 6-shogaol helps to neutralize free radicals and protect against oxidative damage, contributing to its overall neuroprotective effect. Based on earlier studies, these findings are consistent with Stoilova et al., Jeena et al., and Qi et al. [32,33,34].

Neuroinflammation is another factor causing PD. Overexpression of inflammatory cytokines stimulates the degenerative pathway and results in neuronal damage and reduced cerebral blood flow [35]. In earlier investigations, rotenone caused neuronal inflammation, which increased cytokine levels, i.e., IL-1β, TNF-α, and NF-κB, have been connected to improved vulnerability to grow PD after exposure to toxins [36]. By dropping cytokines such as IL-1β, TNF-α, and NF-κB [37]. 6-Shogaol significantly suppresses the expression of these cytokines, likely by inhibiting the activation of NF-κB, a key transcription factor in the inflammatory response. This reduction in inflammation helps protect neurons from the inflammatory damage typically seen in PD (Figure 8A–C). These results agree with reported studies by Jambi et al., Altharawi et al., and Alabi et al. [15,25,38].

6-Shogaol inhibited the rotenone-induced reductions in DA and GABA levels and elevation in MAO-B level, as demonstrated in Figure 9A–C. It has been hypothesized that 6-shogaol potentially suppresses the monoamine oxidase enzyme (MAO-B) in animal brains [16]. These outcomes recommend that 6-shogaol can enhance the dopaminergic activity and the rotenone-treated animals’ motor abilities. MAO-B breaks down DA enzymatically in the brain to produce DOPAC [39]. It is broadly recognized that MAO-B plays a pathogenic role in PD by activating certain toxins and producing free radicals [22]. Additionally, dopaminergic neurodegeneration is caused by the MAO-B metabolism of DA [40]. The 6-shogaol-treated rats restored the abnormal levels of neurotransmitters, increasing DA and GABA and lowering MAO-B. 6-Shogaol may inhibit MAO-B, an enzyme that breaks down dopamine. By inhibiting MAO-B, it could increase the levels of dopamine in the brain. 6-Shogaol binding affinity to GABA receptors which might contribute to its neuroprotective effects by maintaining the balance of excitatory and inhibitory signals in the brain. These results agree with reported studies by Alzarea et al., and Alghamdi et al. [39,40].

The binding energy values of 6-shogaol suggest that it exhibits favorable interactions with AChE and MAO-B, indicating its potential as an inhibitor of these enzymes. Inhibition of AChE and MAO-B has been proposed as a potential therapeutic approach for the treatment of PD. AChE is responsible for the breakdown of Ach, a neurotransmitter involved in motor control. By inhibiting AChE, 6-shogaol may enhance the levels and activity of Ach, leading to improvement in motor symptoms. On the other hand, 6-shogaol exhibits a lower binding affinity to DA and GABA receptors. DA and GABA are neurotransmitters that play important roles in modulating the activity of neurons in the brain. While the weaker binding energies suggest a weaker interaction, it does not necessarily mean that 6-shogaol lacks therapeutic potential with these receptors. It is possible that the 6-shogaol may have additional mechanisms of action or interact with other receptors that influence DA and GABA signaling. In agreement with prior literature, our docking results are consistent [41]. 

Histopathological examination displayed that treatment with 6-shogaol at both doses exerts beneficial consequences on the intellect impairment caused by rotenone as depicted in Figure 10. According to previous studies, rotenone groups showed neuronal degeneration. The current results showed that 6-shogaol improved the neuronal structure and decreased degeneration of neurons resulting in normal histology of the brain [39].

Molecular docking results suggest that 6-shogaol may have potential therapeutic effects on PD in rotenone-induced rats by modulating the levels and actions of various neurotransmitters, especially Ach, and monoamines (Figure 11).

Rotenone groups showed neuronal degeneration and pigmented neurons, which is a previous study by Abdel-Salam et al. [10]. Park et al. explored the anti-neuroinflammatory effects of 6-shogaol, showing that it protected dopaminergic neurons by inhibiting pro-inflammatory mediators like TNF-α and NO in MPP-induced neuroinflammation in DA neurons of PD models. 

New findings involve in this study preventive effects of 6-shogaol on rote-none-induced PD via behavioral paradigms (open field, forced swim, rotarod, catalepsy). The consequences showed that 6-shogaol, which has a naturally occurring enhances antioxidant defenses (GSH, CAT, SOD) and lowers oxidative markers like MDA and nitrite, which are elevated by rotenone. Additionally, 6-shogaol inhibits pro-inflammatory cytokines (IL-1β, NF-κB, TNF-α) and reduces AChE and MAO-B activity, preserving dopamine levels. 6-shogaol also increases CP, an antioxidant protein, and improves neuronal structure, reducing neurodegeneration and improving histopathological cell damage in the brain. This provides a comprehensive understanding of the 6-shogaol potential to protect rodents from the neurodegeneration caused by rotenone. The limitations of this study’s short duration and minimum animal use. Future research is required to confirm the molecular mechanism of 6-shogaol.

## 4. Materials and Methods

### 4.1. Chemicals

Rotenone (Sigma-Aldrich, St. Louis, MO, USA) was employed in the research. The investigative kits for IL-1β, NF-κB, and TNF-α were dignified by the use of a rat enzyme-linked immunosorbent assay (ELISA) kit (Krishgen Biosystems, Mumbai, India).

### 4.2. Animals

Male Wistar rats (10–12 weeks of age; 180 ± 20 g) were acquired from the animal center and research laboratory of T-G Services, Maharashtra, India. Animals were housed and maintained under standard laboratory conditions with ad libitum access to food, water, and environmental enrichment. Rats were familiar with laboratory environments and animals included in the study with no previous procedure. In polyacrylic cages, rats were sustained at a consistent 25 °C temperature and comparative moisture of 45–55% with 12:12 h light/dark cycles. A standard pellet diet and unrestricted access to water were supplied to the animals. The animal ethics committee of the institution (IAEC/TRS/PT/22/18) approved the study’s design, which was conducted as per the ARRIVE guideline.

### 4.3. Research Design

Rats were randomized into five groups (n = 30): Group I (normal control- saline), Group II (rotenone control-subcutaneously with 2 mg/kg rotenone), Group III (6-shogaol per se 20 mg/kg), Group IV and V received 6-shogaol + rotenone (10 and 20 mg/kg) orally. This treatment plan was continued every day for 28 days. On day 29, i.e., 24 h following the final dose, animals were assessed for rotarod test, open field test, forced swim test, and catalepsy. After behavioral assessments, animals were euthanized to obtain brain tissue for biochemical analysis. Brain homogenates were assayed for AChE activity, oxidative stress indicators (malondialdehyde, catalase, superoxide dismutase, glutathione), nitrosative stress, ceruloplasmin concentration, and proinflammatory markers including NF-κB, IL-1β, TNF-α and neurochemical markers (DA, 3,4-dihydroxyphenylacetic acid-DOPAC, gamma-aminobutyric acid-GABA, Monoamine oxidase-B-MAO-B).

### 4.4. Behavioral Test

#### 4.4.1. Open Field Test

The open arena is made up of a big square wooden box that is 50 cm high, 1.2 m long, and divided into 16 squares on the bottom. The core four squares were positioned between the remaining 12 squares beside the walls, which were referred to as the margin squares. Individual rats were left in open fields for five minutes apiece, and their climbing, rearing, and line-crossing behaviors were noted. Climbing occurs once an animal rests its front paws instead of a wall; rearing occurs when both front paws are raised off the ground; and line crossing occurs when all four paws are moved from one square and placed in another. Separate counts were made for crossings between the core and periphery squares [42,43,44].

#### 4.4.2. Forced Swim Test

A forced swim behavioural test evaluates the efficiency of innovative remedies in treating depression [45]. The training period was executed, and rats were permitted to swim in a water-filled container (25 cm wide × 40 cm long × 16 cm high) for 15 min. After 29 days, animals were once more subjected to swim for 5 min to measure immobility time (the duration necessary to obtain complete body immobility with only movement allowed) to continue the head on the water’s surface. Water from the tank was replaced after each animal throughout the test to reduce the influence of odor [46,47].

#### 4.4.3. Rotarod Test

A rotarod behavioral test examined the motor coordination of animals. A rotational bar with a diameter of 7 cm was used to hold rats (speed 25 rpm). The 180-s cut-off period measured the average time it took to descend [48,49].

#### 4.4.4. Catalepsy

Catalepsy was tested per a published protocol with a few modifications [6]. After placing the rats in a standing position, the latencies spent by each limb to take out one of its forelimbs from a 10 cm high bar were calculated [6,50]. Three successive trials were performed to determine the interval of time. For the bar test, 180 s was the highest cut-off time.

### 4.5. Biochemical Evaluations

#### 4.5.1. Brain Tissue Homogenate Preparation

Blood was collected by retro-orbital plexus using small capillary tubes just before sacrificing the rats [51]. The rats were anesthetized with an intraperitoneal injection of ketamine (75 mg/kg) and xylazine (10 mg/kg) and sacrificed by cervical dislocation. After decapitating the animals, the striatum was extracted, placed on ice, weighed, and commingled in phosphate buffer (pH 7.4, 0.1 M). After centrifuging the homogenized at 10,000× *g*, aliquots of the supernatant were isolated for 15 min and utilized for biochemical assessment [49,52,53].

#### 4.5.2. AChE Activity

AChE activity was evaluated employing Ellman’s technique using aliquots from the brain homogenates. The absorbance deviations were instantly dignified at 412 nm. AChE activity was indicated in μmol/g [54,55].

### 4.6. Endogenous Antioxidant Levels

#### 4.6.1. GSH Level

Previously described procedures used to measure GSH. The homogenate was blended with 1.0 mL of centrifuged Ellman reagent and 10% trichloroacetic acid. At a wavelength of 412 nm, the reaction mixture’s absorbance was measured. GSH activity was displayed in mg/g tissue [49,56].

#### 4.6.2. CAT Level

The cuvette was filled with 0.1 mL of tissue supernatant and 1.9 mL of phosphate buffer (50 mM, pH 7.0). The reaction was initiated by adding 1.0 mL of newly formed H_2_O_2_ (30 mM) to the cuvette. At 240 nm wavelength, the absorbance was measured every 10 s for 1 min. One minute of CAT was elucidated as the number of enzymes requisite to molder one mmol of peroxide per minute at 25 °C with PH 7.0. CAT was measured as μmole/H_2_O_2_ decomposed/min/mg activity [49,57].

#### 4.6.3. SOD Level

An amount of 0.2 mL of brain homogenate supernatant was blended with 0.8 mL of glycine buffer (50 mM, pH 10.4) together with 0.02 mL of epinephrine; after adding it to start the reaction, it was set aside for five minutes. At 480 nm, the alteration in optical density was determined and regularized to a blank reagent per minute. The SOD activity was represented in unit SOD activity (mg/protein) [49,58]. SOD activity was measured in SOD Units/mg tissue.

### 4.7. Oxidative and Nitrative Stress Markers

#### 4.7.1. MDA Level

The MDA level was quantified in brain homogenates using the Wills method (1996). Thiobarbituric acid (TBA) responsive ingredients react with MDA to generate TBA-MDA metabolite. The peak absorbance was estimated spectrophotometrically at 532 nm, and activity was revealed as nmol/g tissue [10,59].

#### 4.7.2. Nitric Oxide Level

Griess reagent was employed to find out the nitrite level in the brain homogenate [60]. Nitric oxide is a stable product of nitrite. A nitrite radical is used as an indicator to produce nitric oxide. At 546 nm wavelength, the absorbance was expressed [10], and the activity was represented in mmol/g tissue [61,62].

#### 4.7.3. Ceruloplasmin (CP) Level

CP plays a role in iron metabolism as a ferroxidase. Mutations in the ceruloplasmin gene have confirmed ceruloplasmin’s role in brain iron metabolism. An ELISA technique was used to estimate CP based on instructions provided by the producer [63]. CP activity was measured in CP ng/g tissue [64].

### 4.8. Proinflammatory Markers

ELISA, TNF-α, IL-1β, and NF-κB were measured following standard procedure. The separated proteins from the homogenate were pipetted in an antibody-coated ELISA plate. The number of cytokines was determined using the standard assay procedure [15,49]. 

### 4.9. Neurotransmitters Markers

To estimate the level of neurotransmitters like DA, GABA, and monoamine oxidase B (MAO-B), they were measured using HPLC [15].

### 4.10. Histopathology of the Brain

According to protocol, brain tissues were secured in 10% buffered formalin, processed in graded ethanol, and then immersed in paraffin. Hematoxylin and eosin (H and E) were employed to stain sections of 5 µm thickness for histopathological analysis under a light microscope.

### 4.11. Molecular Docking

Molecular docking was employed to evaluate the binding affinities of the selected compounds to the target proteins. These interactions are essential for elucidating the potential therapeutic efficacy of these compounds. Protein and Ligand Preparation: Crystallographic structures of AChE (PDB ID: 7XN1), DA (PDB ID: 6CM4), GABA (PDB ID: 6CDU), and MAO-B (PDB ID: 2V5Z) were obtained from the RCSB Protein Data Bank. The proteins underwent structural optimization and minimization using CHIMERA v1.16, with the removal of nonstandard residues, water molecules, cocrystal ligands, and unnecessary chains. Ligands, including 6-shogaol retrieved from ChemSpider, were imported into MarvinSketch for 2D and 3D cleaning. MMFF94 force field was applied for minimization, and the lowest energy conformer in the MOL2 format was chosen for further analysis. Grid Generation: AutoDock Tools 1.5.65, Chimera 1.112, and Maestro Version 12.7.1619 were used for grid generation and validation. Grid parameters were determined based on the co-crystal ligand orientation or CASTp server for proteins in the Apo state. Molecular Docking: Ligand and protein structures were converted to pdbqt format using in-house Bash scripts with AUTODOCK Tools 1.5.65. AutoDock Vina 1.2.57.8 was employed for docking studies with a grid spacing of 0.375 Å. The grid box was centered on the target’s active site, and specific grid parameters were set for each protein. CPU was configured for 23, exhaustiveness for 32, number of modes for 9, and energy range for 3. After docking, protein-ligand complexes were visualized using Biovia Discovery Studio Visualizer, LigPlot v1.4.510, and Maestro 12.79 by generating 2D and 3D images. Detailed interactions between proteins and ligands were identified through the PLIP server, and results are tabulated for reference.

### 4.12. Statistics Analysis

All statistics are accessible as mean ± standard error (S.E.M.). Utilizing GraphPad Prism software (Version 8.0.2.), we performed a One-Way Analysis of Variance (ANOVA) followed by a Tukey post hoc comparison test, and the consequence level was measured at *p* < 0.01.

## 5. Conclusions

The study concluded that 6-shogaol produced an anti-Parkinson effect in the rotenone-induced paradigm. 6-Shogaol lessens rotenone, causing behavioral and biochemical irregularity in rats by dropping inflammatory reactions, oxidative damage, and progression through its antioxidative mechanism. Treatment with 6-shogaol resulted in a notable enhancement of AChE activity and ACh levels, leading to improved motor function. Additionally, the 6-shogaol effectively restored aberrant endogenous antioxidant levels, including GSH, CAT, and SOD. Furthermore, 6-shogaol mitigated oxidative and nitrative stress markers, indicative of its antioxidant properties. Concurrently, it exhibited anti-inflammatory effects by inhibiting the expression of inflammatory mediators such as IL-1β, TNF-α, and NF-κB and restored the catecholamines markers. The molecular docking results confirm that the 6-shogaol strongly binds with the binding site of AChE (−8.214 kcal/mol) and MAO-B (8.133 kcal/mol). Overall, these findings suggest that 6-shogaol possesses promising neuroprotective and anti-inflammatory properties. The brevity of the experimental timeline and the limited number of animals utilized may have constrained the depth of exploration into the research challenges encountered. The study was circumscribed by the use of smaller sample sizes and a restricted scope of molecular analyses, encompassing techniques such as immunohistochemistry and Western blotting. Moreover, the investigation was limited by the reliance on a single model and the absence of a broader evaluation of gene and protein expression, including the use of genetic models. Future investigations are necessary to elucidate the precise molecular mechanisms underlying the effects of 6-shogaol, as these findings provide a critical foundation for the development of novel therapeutic strategies. With further validation through a well-designed clinical study, 6-shogaol holds promise as a potential therapeutic candidate for Parkinson’s disease.

## Figures and Tables

**Table 1:** Docking Score and intermolecular interactions of ligand 6-Shogaol Proteins AChE (7XN1), Dopamine (6CM4), GABA (6CDU), MAO-B (2V5Z) using LigPlot v1.4.5, PLIP server, Maestro V12.8 and Biovia Discovery studio visualizer.

Sr. No	Name of Compound	Binding Energy	Type of Interaction	Residue ID	Distance
1	7xn1_6-Shogaol	−8.214	Hydrophobic Interactions	ASP74A	3.51
				TRP86A	3.66
				TRP86A	3.65
				TRP86A	3.99
				TYR337A	3.97
				TYR337A	3.94
			Hydrogen Bonds	TRP86A	2.09
				TYR337A	3.52
2	6cm4_6-Shogaol	−7.396	Hydrophobic Interactions	VAL91A	3.75
				LEU94A	3.98
				TRP100A	3.83
				PHE110A	3.77
				ILE184A	3.42
				PHE189A	3.76
				PHE189A	3.98
				PHE389A	3.76
				PHE389A	3.43
				PHE390A	3.63
				THR412A	3.99
			Hydrogen Bonds	SER193A	2.47
				SER197A	2.81
				HIS393A	2.45
			π-Stacking	TRP386A	5.34
3	6CDU_6-Shogaol	−6.189	Hydrophobic Interactions	ILE239A	3.63
				VAL243A	3.77
				VAL243A	3.48
				TRP246A	3.3
				TRP246A	3.83
				ILE302B	3.88
				THR306B	3.53
				TYR309B	3.7
				PHE310B	3.68
			Hydrogen Bonds	TRP246A	2.22
				THR306B	3.65
4	2v5z_6-Shogaol	−8.133	Hydrophobic Interactions	LEU171A	3.74
				LEU171A	3.75
				GLN206A	4
				TYR326A	3.84
				PHE343A	3.33
				TYR398A	3.39
				TYR398A	3.7
				TYR398A	3.84
				TYR398A	3.6
				TYR435A	3.67
				TYR435A	3.61
			Hydrogen Bonds	ILE198A	2.24

## Data Availability

The original contributions presented in the study are included in the article, further inquiries can be directed to the corresponding authors.
